# Commentary on: Calpain-2 participates in the process of calpain-1 inactivation

**DOI:** 10.1042/BSR20203690

**Published:** 2021-03-31

**Authors:** Raniki Kumari, Tushar Kanti Maiti

**Affiliations:** 1 Functional Proteomics Laboratory, Regional Centre for Biotechnology (RCB), NCR Biotech Science Cluster, Faridabad 121001, India; 2 School of Biotechnology, Kalinga Institute of Industrial Technology (KIIT), Bhubaneswar, Odisha 751024, India

**Keywords:** apoptosis, calpain-1, Calpain-2

## Abstract

Calpain belongs to the calcium-dependent non-lysosomal cysteine protease. Calpain-1 (C1) and calpain-2 (C2) expression are ubiquitous in mammals and an important mediator of the action of calcium. Specific substrate cleavage by C1 and C2 is critical for several calcium-dependent cellular pathways including neuronal function, muscle contraction, signal transduction, cell differentiation, proliferation, and apoptosis. Research suggests that C1 and C2 perform similar functions due to their structurally highly similar isoforms. Increasing evidence suggests that C1 and C2 carry out their specific function *in vivo.* A recent paper published by Shinkai-Ouchi et al. (*Bioscience Reports* (2020) **40**, DOI: 10.1042/BSR20200552) elucidated the mechanism to differentiate the function of each calpain with respect to the efficiency and longevity for proteolysis after activation. Further, the study represented that C1 and C2 do not synergistically perform their work* in vitro*. On the other hand, the activity of C1 is reduced in presence of C2. This insight establishes the platform for future studies to examine how C2 regulates the C1 for substrate proteolysis.

## Introduction

Calpains are intracellular calcium-dependent cysteine proteases that were reported over 30 years ago. The classical calpains are made up of heterodimers composed of a large 78–80-kDa catalytic subunit and a common 29-kDa small regulatory subunit [1]. Calpain-1 (C1) and calpain-2 (C2) are the most studied members of the calpain family due to their ubiquitous expression in mammals [2]. C1 and C2 are termed µ-calpain and m-calpain, depending upon the need for a concentration of intracellular Ca^2+^ for activation, in the micromolar and millimolar ranges, respectively [3]. The C1 contributes to more physical processes than C2 due to the low level of Ca^2+^ requirement, which is easy to attain *in vivo* [4]. The elevation of intracellular Ca^2+^ concentration is mandatory for the activation of calpain. However, other factors may also activate calpain via signaling pathways such as the extracellular signal-regulated kinases/mitogen-activated protein kinases signaling pathway [4]. Many proteins are the substrate of calpains, including myocardial proteins, transcription factors, signal transduction protein, cytoskeleton, and plasma membrane-associated proteins. It indicates that calpains play a regulatory role in a variety of cellular functions that are dependent on the Ca^2+^ [5].

C1 and C2 isoforms have long been considered that they work in a complementary manner due to their ubiquitous expression and dependence on Ca^2+.^ They are structurally highly similar isoforms of calpain. However, studies have revealed that each calpain species performs a specific function *in vivo*.

In their work, Shinkai-Ouchi et al. set out the experiments and tried to understand the mechanism to differentiate between C1 and C2, as well as to elucidate the mechanism relevance to their substrate proteolytic activity [6]. Using cardiac troponin T (TnT), as a substrate *in vitro* in combination with iTRAQ-based mass spectrometry, they have shown that proteolytic activity of C1 lasts longer than C2. The previous study has shown that calpain undergoes autolysis parallel with substrate proteolysis. Authors showed that, during TnT digestion, both C1 and C2 autoproteolyzed. However, C2 has a stronger tendency to autolyze at multiple sites. After analyzing the autolytic fragments of C1 and C2, they revealed that C1 and C2 differed in terms of the complexity of their autolytic products and found that C2 undergoes more heterogeneous and aggressive proteolysis at consecutive peptide bonds. Further, they analyzed the reciprocal proteolysis of C1 and C2 and observed that C2 proteolyzes C1 as a substrate and cleavage occurs between 126 and 127 amino acids of C1. Further, during C1 activation, autolysis occurred in the N-terminal of C1 which produced catalytically active 78- and 76-kDa fragments. The identified peptide fragments indicated that C2 further trims the N-terminal region of 76-kDa C1 fragment and destabilizes the autolyzed form of C1.

As the C2 reduced the activity of C1 by destabilizing the autolyzed fragments, it is important to examine the proteolytic activity of C1 for its substrate. To prove this, the authors have demonstrated that in presence of C2, the proteolytic activity of C1 towards TnT is reduced. Further, they elucidated the effect of C2 on activation-associated autolysis of C1 in a complex system such as different cell lines by knocking down *C1* or *C2* genes. Authors observed that C2 recognizes C1 as a substrate in the cell lysate and enhances the decay of C1.

Calpains get activated in several cell systems such as C1 activation on platelet activation by thrombin [7], in neuronal cells after hypoxia–ischemia–reperfusion to start the apoptosis cascade, in cultured cortical neurons exposed to excitotoxic agent including NMDA in SH-SY5Y cells stimulated with calcium ionophore [8]. C1 activation in endothelial cells upon adhesion to fibronectin, in adhered smooth muscle cells treated with a fragment of collagen I [9]. In contrast with C1, C2 performs its critical role in the terminal differentiation of many cell systems including monocytic differentiation of K562 cells, HaCaT keratinocyte differentiation, and the differentiation of rodent osteoblasts ROB-C26 and MC3T3-E1. Apart from this, calpains are involved in apoptosis. Calpains inhibits the ability of cytochrome *c* to activate the caspase cascade [9]. Several studies suggest that C1 and C2 have a critical role in various cellular processes.

**Figure 1:**
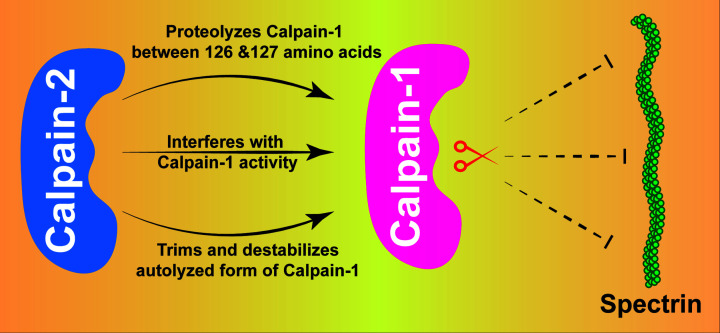
Schematic illustration of the modulation of Calpain-1 activity by Calpain-2 Calpain-2 proteolyzes, interferes with the activity of Calpain-1, and destabilizes autolyzed form of Calpain-1. Subsequently, Calpain-1 compromises to proteolyze its substrate.

Shinkai-Ouchi et al. showed mechanistic insight into proteolytic cleavage activity of C1 and C2 [6]. Down-regulation of C1 by C2 provides novel mechanistic insight into regulation of C1 (Figure 1). These findings provide a platform for establishing C1 as a therapeutic target in near future.

## Abbreviations

C1: Calpain-1
C2: Calpain-2
iTRAQ: Isobaric Tags for Relative and Absolute Quantitation
NMDA: N-Methyl-D-Aspartate
TnT: Troponin T
